# Psychosocial Working Conditions and Cognitive Complaints among Swedish Employees

**DOI:** 10.1371/journal.pone.0060637

**Published:** 2013-04-01

**Authors:** Cecilia U. D. Stenfors, Linda Magnusson Hanson, Gabriel Oxenstierna, Töres Theorell, Lars-Göran Nilsson

**Affiliations:** 1 Department of Psychology, Stockholm University, Stockholm, Sweden; 2 Stress Research Institute, Stockholm University, Stockholm, Sweden; 3 Department of Public Health Sciences, Karolinska Institute, Stockholm, Sweden; 4 School of Technology and Health, Royal Institute of Technology, Stockholm, Sweden; University of Pennsylvania, United States of America

## Abstract

**Background:**

Cognitive complaints involving problems with concentration, memory, decision-making and thinking are relatively common in the work force. The sensitivity of both subjective and objective cognitive functioning to common psychiatric conditions, stress levels and to cognitive load makes it plausible that psychosocial working conditions play a role in cognitive complaints. Thus, this study aimed to test the associations between psychosocial work factors and cognitive complaints in nationally representative samples of the Swedish work force. Cross-sectional (n = 9751) and prospective (n = 3644; two time points two years apart) sequential multiple regression analyses were run, adjusting for general confounders, depressive- and sleeping problems. Additional prospective analyses were run adjusting for baseline cognitive complaints.

**Cross-sectional results:**

High quantitative demands, information and communication technology (ICT) demands, underqualification and conflicts were positively associated with cognitive complaints, while social support, good resources at work and overqualification were negatively associated with cognitive complaints in all models. Skill discretion and decision authority were weakly associated with cognitive complaints. Conflicts were more strongly associated with cognitive complaints in women than in men, after adjustment for general confounders.

**Prospective results:**

Quantitative job demands, ICT demands and underqualification were positively associated with future cognitive complaints in all models, including when adjusted for baseline cognitive complaints. Decision authority was weakly positively associated with future cognitive complaints, only after adjustment for depressive- and sleeping problems respectively. Social support was negatively associated with future cognitive complaints after adjustment for general confounders and baseline cognitive complaints. Skill discretion and resources were negatively associated with future cognitive complaints after adjustment for general confounders. The associations between quantitative demands and future cognitive complaints were stronger in women.

**Discussion/Conclusions:**

The findings indicate that psychosocial working conditions should be taken into account when considering cognitive complaints among employees.

## Introduction

Modern working life has been described as more intense than in the past due to greater competition between companies and rapidly changing conditions at the workplace. The development of information and communication technologies has also intensified work by providing new possibilities and demands for communication and information management [1], [2]. From this follows that a high level of cognitive functioning has become ever more vital in order to manage work and maintain ability to work. At the same time, these new working conditions may cause cognitive problems for some individuals. The role that psychosocial work factors might have on cognitive functioning has been scarcely studied but this is an increasingly important question as work life moves toward being more information intensive and cognitively demanding.

The aim of the present study was thus to explore relations between psychosocial work factors and reported cognitive problems. Specifically, the aim was to study associations with some modern work factors, which may be seen as either protecting or reducing cognitive resources and affecting the incidence and development of experienced cognitive difficulties in the Swedish working population. The cognitive problems that have been measured in the present study include self-rated ability to focus attention, to remember, to make decisions, and to think clearly. These have also been identified as common cognitive symptoms of stress [3]. We will refer to self-perceived problems with these abilities as cognitive complaints.

Common conditions where cognitive complaints are particularly prevalent in the adult population, such as stress related conditions (burnout or exhaustion) and depression, are often associated with reduced work ability [4], [5], [6], as well as deficits in objectively measured cognitive functioning [7], [8], [9], [10], [11], [12], [13], [14]. These disorders are among the most common causes of sick-leave in Sweden [15] and cognitive problems have been reported as a key obstacle to manage work tasks [4], [5]. Cognitive symptoms that have been described in the period preceding job-stress related exhaustion are a diminished capacity to fulfil work tasks, deterioration in cognitive functioning in terms of concentration and decision making, failing memory, and black-outs [4].

Studies on non-clinical healthy samples have also shown that both chronic and acute stress can impair cognitive functioning [16], [17], [18], [19].

Such findings make relationships between aversive working conditions and cognitive complaints the more expectable.

However, the relationship between cognitive complaints and measures of actual cognitive functioning have differed across studies, sometimes showing a stronger relationship to negative affective conditions or states, when studied at the group level. Most of these studies on the relation between cognitive complaints and cognitive functioning have been made on elderly [20], [21], [22], [23], [24], [25], [26], [27], [28], [29], [30], [31], while population studies on non-elderly adults are few with mixed results [32], [33], [34], [35], [36]. This in addition to methodological issues regarding measures of cognitive functioning in these studies make it difficult to draw any clear conclusions.

Thus, while it is hard to draw any firm general conclusions regarding the relationship between cognitive complaints and measures of cognitive functioning among working-age adults, cognitive complaints seem to be an important aspect of well-being, psychological health and possibly also an indicator of day-to-day cognitive capacity at the individual level. Thus, this merits further study.

One of the leading work environment models is the demand-control-support model [37]. This model predicts that high psychological demands, low decision authority, low skill discretion and low social support at work will be detrimental to health. The model and its subcomponents have been extensively studied and show associations with various health outcomes including psychological health and well-being [38], [39], [40], [41] as well as poorer learning outcomes in call centre employees [42]. Job strain (high demands and low control) has also been related to lowered cognitive function in civil servants in London (before adjustment for employment grade) [43], and more cognitive complaints in Danish employees [44], [45], [46]. The Danish studies showed a positive relationship between high skill discretion and cognitive complaints, suggesting that exceedingly high demands for learning and skill may turn from being beneficial and health promoting into being adverse. Studies of Danish employees also found cognitive complaints to be associated with conflicts at work, as well as exposure to violence, role conflicts, and lack of role clarity, -recognition, -predictability, -influence, -social support, -meaning at work, and low decision authority [44], [45], [46]. Other work factors that have been related to psychological health include information and communication technology (ICT) usage and demands [47] which have also been observed to have negative effects on performance of cognitively demanding work tasks [48]. Emotional demands have also been shown to potentially affect cognitive complaints negatively [49].

According to the Job Demands-Resource model of work and health [50], [51], [52], strain and stress from work depends on the balance between job demands and the job resources that are available to meet those demands. Accordingly, additional types of resources to those in the demand-control-support model are relevant for psychological health, including having sufficient material resources [53] as well as one's own resources in terms of qualification for the job. Hence these were also included in the current study.

Given the hitherto few and sometimes contradictory studies on working conditions and cognitive function or cognitive symptoms which have been mostly carried out on specific groups, general conclusions on this subject cannot be drawn. The aim of the present study was thus to study both cross-sectional and prospective relations between psychosocial work factors and reported cognitive complaints in a large sample that is approximately nationally representative of the Swedish work force, including all sectors and occupations.

Based on previous studies on work environment factors and psychological health, the following associations between work environment factors and cognitive complaints were hypothesized: High quantitative demands, ICT demands, emotional demands, conflicts and being underqualified would be positively associated with cognitive complaints. High decision authority, social support, resources, and possibly being overqualified, would be negatively associated with cognitive complaints. A hypothesis regarding skill discretion was difficult to formulate since studies on different samples have provided conflicting findings.

In addition we aimed to study the extent to which depressive symptoms and sleeping problems play a role in the relationships between work factors and cognitive complaints, since depressive symptoms and sleeping problems are both related to cognitive complaints [14], [54] and to working conditions[39], [41], [55], [56], [57].

Lastly, since cognitive complaints have been observed to be more prevalent among women in the work force, like other related conditions such as exhaustion and depression [58] and since gender differences in the associations between working conditions and mental health symptoms have been observed previously (e.g. [40], [59]), we also aimed to investigate the presence of gender differences in the hypothesized relationships.

## Methods

### Study participants

Participants were recruited from the Swedish Longitudinal Occupational Survey of Health (SLOSH) - a longitudinal study of work environment and health among Swedish employees that is conducted biennially. The first wave of data was collected in 2006 (i.e. T1) and the second wave in 2008 (i.e. T2) by inviting participants from the Swedish Work Environment Surveys (SWES: conducted in 2003 and 2005) to answer more detailed self-completion questionnaires. SWES is conducted biennially by Statistics Sweden and is a stratified random sample of the respondents to the Swedish Labor Force Survey aged 16–64 years.

In 2006, respondents to SWES 2003 were invited to participate in SLOSH wave I with 5985 respondents (65% response rate), out of whom 5141 were “gainfully employed”- defined as working at least 30% on average during the past three months. Respondents to both SWES 2003 and SWES 2005 were invited to participate in SLOSH 2008 with a total of 11,441 respondents (61% response rate), out of whom 9,751 (85%) were gainfully employed.

Comparisons between the original SWES samples and those who responded to SLOSH showed that women, older subjects (aged 50+) and married/cohabiting subjects as well as men and women with high education were overrepresented among responders. There were no differences regarding country of birth or citizenship.

#### Cross-sectional analyses

In the current study were conducted on those respondents to SLOSH wave II (2008) who were gainfully employed (n = 9751).

Subjects with incomplete data on any of the variables of interest were excluded in the main analyses, resulting in a final cross sectional study sample of 8362 participants. Thus 14.3% cases were missing from the cross-sectional study sample in the regression model due to missing data on some measures.

Descriptive statistics for the sample participants are shown in table S1.

#### Longitudinal analyses

Were performed on the prospective study sample including those who participated in both wave I (2006) and wave II (2008) (n = 4690, 78% of all participants in wave I) and were gainfully employed at both occasions (n = 3644; 46.7% men, 53.3% women).

As it is based on the SWES 2003, the prospective study sample was approximately representative of the Swedish working population in 2003. Subjects with incomplete data on any of the variables of interest were excluded, resulting in a prospective study sample of 3264 individuals. Thus, 10.4% of the cases were missing from the prospective study sample in the regression models due to missing data.

Compared to all participants in SWES 2003 (n = 9214), a higher proportion of the respondents included in the prospective study sample were in the age range 40–59 in 2003, and a higher proportion had university education. However, the gender distribution was virtually the same, as were the mean scores on demand, control, and support proxy measures from SWES 2003. For more details and flowchart of the prospective study sample, see [60].

### Questionnaire measures

#### Demand, control and support

Quantitative psychological job demands (having to work fast,-intensively, -with too much effort, not having enough time and having conflicting demands), Skill discretion (requirements of skilfulness, -learning new things and repetitiveness of work tasks), Decision authority (deciding how and what to do in/at work) and Social support (e.g. getting along well with colleagues) are derived from the Demand-Control-support model and were measured by five, four, two, and six items (scale 1–4) respectively, from the Swedish version of the Job-Content Questionnaire [37], [61].

#### Information and communication technology (ICT) demands

Were measured by six items (scale 1–5) [62], largely based on work by Johansson-Hidén et al. [63]). These cover questions about the extent of interruptions by- and demands of having to answer too many e-mails and telephone calls, technical problems with ICT and the role that ICT might play in eroding boundaries between work and leisure, i.e. always being ‘on call’.

#### Emotional demands

Were measured by two questions about frequency of the demand to be empathic at work and if work puts you in emotionally disturbing situations (scale 1–4) [64], [65].

#### Material resources

At work were measured by three questions about resources related to economics, personnel, and equipment (scale 1–4) at wave II. Material resources were measured by a single item at wave I (scale No/Yes).

#### Qualification level

The perceived level of qualification for the job was measured by a single question with five response alternatives that were recoded into 3 categories: Under-qualified, Qualified, and Overqualified.

#### Conflicts

Involvement in any conflict at work in the past two years with superiors, colleagues or clients was measured by three yes/no questions. Current ongoing involvement in any conflict was measured by a single item (yes/no). These four items were combined into three categories: No conflicts, Terminated Conflicts, and Ongoing Conflicts.

#### Cognitive complaints

Were measured by four questions about difficulties during the past 3 months with concentration, memory, decision-making and ability to think clearly, all on a scale from 1–5/’Never’-‘Always’. The scale was adopted from the Copenhagen Psychosocial Questionnaire [65] and has its origin in The Stress Profile questionnaire [3]. An index was created from the mean score of the four questions, where a high score indicated a high degree of cognitive complaints.

#### Depressive symptoms

During the past week were measured by six items (scale 1–5) selected from the Hopkins Symptom Checklist depression subscale (SCL-90, [66]), using clinical validity as primary selection criteria [67], [68]. Items cover symptoms such as feeling blue, feeling no interest in things and worrying too much about things.

#### Sleeping problems

Were measured with the established and validated measures Disturbed sleep index, reflecting a lack of sleep continuity, and the Awakening index, reflecting feelings of being insufficiently restored during the past 3 months. Dichotomised variables were used to indicate the presence or absence of sleep disturbances and awakening problems (i.e. not feeling sufficiently restored after sleeping), based on four and three items respectively [56], [69], [70].

#### Other potential confounders considered

Age, Sex, Educational level ('No upper secondary school', ‘Upper secondary school’, ‘Undergraduate studies <2 years’, ‘Undergraduate studies ≥2 years); Yearly income; Alcohol habits/binge drinking (How often do you drink 6 glasses or more at one occasion? ‘Never’, ‘Less than once a month’, ‘Every month’, ‘Every week or more’), and the presence of chronic and/or serious Cardiovascular disease (CVD) or Psychiatric illness. All covariates included in the analyses were significantly (p≤0.05) correlated with the dependent variable as well as to one or more of the predictor variables at the bivariate level. BMI and diabetes were also measured but were not adjusted for in the regression analyses since these were not significantly associated to the outcome measure cognitive complaints. These confounders included in the analyses will be referred to as “general confounders”.

All the questionnaire measures described above were used in the cross-sectional and prospective analyses and were collected in wave I and wave II. Only conflicts were excluded in the prospective analyses due to the temporal aspect in the question, asking about conflicts during the past two years.

Indices based on mean scores on individual items were used, where applicable, in subsequent data analyses. High values on any scale in the analyses always indicate a high level of the construct, e.g. high demands, high resources, high support. For all indices used, up to 25% missing item responses were allowed within the same index.

Descriptive statistics for the main study measures in the cross-sectional sample and correlations of each measure from wave I/T1 to wave II/T2 are shown in table S2. Crude correlations between all measures are shown in table S3.

### Data analysis

Data analyses were performed using SPSS software version 19.0. Two types of analyses were performed, cross-sectional and prospective.

#### Cross-sectional analyses

All measures used in the cross-sectional analyses were derived from data collected in 2008, corresponding to T2 in the prospective analyses.

No variables had 5% or more missing values. Thus, missing data on any of the measures used in the respective statistical models were deleted list-wise without further attrition analysis.

Correlations between psychosocial work factors were generally low (r≤0.3) but some of medium (0.3<r≤0.5) size (see table S3). Thus, none of the psychosocial work factors included in the analyses was statistically redundant.

Sequential multiple regression analyses were then performed entering the cognitive complaints index score as the outcome measure and continuous and categorical (dummy coded) psychosocial work factors as predictors in model 1. Adjustment was additionally made for general confounders in model 2, for depressive symptoms in model 3, and for sleeping problems in model 4.

#### Prospective analyses

In the corresponding prospective analyses, measures from T1 were used as predictors for the outcome measure of cognitive complaints at T2.

Each sequence of the prospective regression analyses were also performed with additional adjustment for baseline cognitive complaints (at T1), in order to test if there were prospective relationships between work factors and cognitive complaints that were independent of previous levels of cognitive complaints. As there are limitations both in performing analyses that do not take the baseline levels of the outcome measure into account as well as those that do adjust for the baseline level of the outcome, both types of analyses were performed and all these results are presented in table S5. No baseline adjustment for the outcome measure may inflate the prospective relationships between the predictors and outcome. It may partially reflect the cross-sectional relationships when both the predictors and the outcome measure are auto-correlated over time, which is the case here. Baseline adjustment of the outcome measure on the other hand may induce over-adjustment of the prospective relationships. Thus, the results from these different analyses should be interpreted with these potential caveats in mind.

Interactions between gender and psychosocial work factors were tested by entering the interaction term with gender for each work factor in a first step together with gender and the work factor measures. Tests of gender interactions with psychosocial work factors were performed using SAS software.

Analyses were also performed entering work factors, depressive symptoms and sleeping problems in the reversed order of entry to see how much variance in cognitive complaints could be explained by the work factors alone, independently of depressive symptoms and sleeping problems, when added to the equation in the last step/model.

### Ethics statement

The study has been approved by the Regional Research Ethics Board in Stockholm (Dnr 2006/158-31; Dnr 2008/240-32). All study participants have given their informed consent. Data were analyzed anonymously.

## Results

### Cross-sectional results

Table S4 presents standardized β coefficients, significance levels and adjusted R^2^ from the sequential multiple regressions testing the cross-sectional relationship between psychosocial work factors and cognitive complaints (n = 8362). Thus, reported coefficients represent how many standard deviations of change in cognitive complaints that is associated with an increase of one standard deviation in the respective predictor variable.

Quantitative demands, ICT demands, perceived underqualification, ongoing- and terminated conflicts were positively associated with cognitive complaints, while social support, good resources at work and overqualification were negatively associated with cognitive complaints, even after adjusting for confounders, depressive symptoms and sleeping problems. Skill discretion and decision authority were also negatively associated with cognitive complaints, the former after adjustment for general confounders and the latter only without any adjustments.

A significant gender interaction was seen in the model adjusted for general confounders, in which ongoing and finished conflicts were more strongly associated with cognitive complaints in women (finished conflicts: β = 0.082, p<0.001; ongoing conflicts: β = 0.092, p<0.001) than in men (finished conflicts: β = 0.035, p<0.05; ongoing conflicts: β = 0.047, p<0.01). This gender difference disappeared after adjustment for depressive symptoms, as the association between conflicts and cognitive complaints then decreased among women.

The prevalence of cognitive complaints when defined as an average of at least one symptom being experienced at least “often” and the remaining three symptoms at least “sometimes” (corresponding to a score of 3.25–5, representing the top decile of the sample distribution) was 8.5% for men, and 13.2% for women.

Depressive symptoms were strongly associated with cognitive complaints and sleeping problems were less strongly associated with cognitive complaints.

Work factors explained 19.9% of the variability (adjusted R^2^) in cognitive complaints, while sequentially adding confounders, depressive symptoms and in the last step sleeping problems increased the explained variance to 23.8, 42.0 and 43.3%, respectively.

When entering the independent variables in the reverse sequential order, adding work factors in the last step, after depressive and sleeping problems, raised the adjusted R^2^ from 0.39 to 0.43.

### Prospective results

Table S5 presents standardized β coefficients, significance levels and adjusted R^2^ from the sequential multiple regressions testing the prospective relationships between psychosocial work factors and cognitive complaints (n = 3264) without and with additional adjustment for baseline cognitive complaints.

Quantitative job demands, ICT demands and perceived underqualification were positively associated with future cognitive complaints, even after adjustments for confounders, depressive and sleeping problems, as well as baseline cognitive complaints. Decision authority was weakly positively associated with future cognitive complaints, only after adjustment for depressive and sleeping problems respectively. Social support was negatively associated with future cognitive complaints after adjustment for confounders and baseline cognitive complaints, and significant when adjusting for depressive symptoms and sleeping problems without adjusting for baseline cognitive complaints. Skill discretion and resources were also negatively associated with future cognitive complaints after adjustment for confounders.

Depressive symptoms at T1 were highly associated to future cognitive complaints at T2, adjusted for all other variables in the models.

A significant gender interaction was seen in that the association between quantitative demands and future cognitive complaints was stronger in women than in men, in all models, and also when including baseline cognitive complaints. Only in women were quantitative demands highly significantly and clearly positively associated with future cognitive complaints after adjustment for baseline cognitive complaints.

The results for quantitative demands and cognitive complaints after adjusting for general confounders were: for women, β = 0.195, p<0.001; and for men, β = 0.099, p<0.001. The corresponding results when adjusting additionally for depressive symptoms and sleeping problems were: for women, β = 0.114, p<0.001; and for men, β = 0.045, not significant (p = 0.083). When adjusting for baseline cognitive complaints in addition to general confounders, depressive symptoms and sleeping problems, the estimates were: for women, β = 0.072, p<0.001; and for men, β = 0.003, not significant (p = 0.899).

Work factors explained 12.1% of the variance in cognitive complaints, while sequentially adding confounding factors, depressive symptoms, and in the last step sleeping problems, increased the explained variance to 14.4, 30.6 and 31.0%, respectively. Entering work factors in the last step, after entering depressive symptoms and sleeping problems, only raised adjusted R^2^ from 0.29 to 0.31.

## Discussion

The present study of cognitive complaints in nationally representative samples of gainfully employed Swedes showed that psychosocial work factors were associated with cognitive complaints primarily cross-sectionally, and to some extent also prospectively. The most robust independent cross-sectional predictors were high quantitative demands, ICT demands, perceived underqualification, and conflicts, which were positively associated with cognitive complaints, and social support, good resources at work and overqualification, which were negatively associated with cognitive complaints, even after adjusting for confounders, depressive symptoms and sleeping problems. Small but significant negative associations were also seen for skill discretion after adjustment for general confounders and for decision authority unadjusted.

The most robust independent predictors of cognitive complaints prospectively were high quantitative job demands, ICT demands and perceived underqualification. These were positively associated with future cognitive complaints, when adjusting for confounders, depressive symptoms, sleeping problems and baseline cognitive complaints. This suggests that these factors may be causes of future cognitive complaints independently of previous low mood, sleeping problems and cognitive complaints.

Decision authority was weakly positively associated with future cognitive complaints, only after adjustment for depressive and sleeping problems respectively. Social support was negatively associated with future cognitive complaints after adjustment for confounders, depressive and sleeping problems respectively. In the models adjusted for baseline cognitive complaints, social support remained a significant predictor prior to adjustment for depressive and sleeping problems. Social support may thus cause future cognitive complaints independently of previous cognitive complaints, while depression is also a potential mediator, which accords with previous findings of social support as an important predictor of depressive symptoms in SLOSH [68]. Skill discretion and resources were also weakly negatively associated with future cognitive complaints after adjustment for general confounders. Thus, skill discretion is a resource rather than a stressor in the general working population when it comes to cognitive complaints, unlike the positive association observed in Danish employees between high skill discretion and high cognitive complaints cross-sectionally [44].

The results are consistent with previous cross-sectional findings of negative associations between cognitive complaints and quantitative demands (though only in women), conflicts, and decision authority (though only in men) in Danish employees [44] and with prospective associations between cognitive complaints, quantitative demands and social support (from management) in Danish knowledge workers before adjustment for baseline cognitive complaints [46]. The presence of the prospective associations also after adjustment for baseline cognitive complaints in the present study could be due to differences in sample characteristics, here including all occupational groups and being larger in size.

Current results are also consistent with studies showing that quantitative demands predict the development of other future psychological health symptoms [38] and that objective measures of high demands are prospectively related to increased risk of depression [55]. Associations with prior conflicts are also consistent with previous findings that conflicts at work are associated with negative effects on affect, cognitive task performance [71] and group performance [72]. However, some types of conflicts (i.e. task conflict) may also be constructive under certain conditions (e.g. of trust, respect, similar values among co-workers) [73], [74]. Furthermore, ICT stress involving information overload and interruptions has been extensively associated with declines in cognitive work task performance [48] in smaller samples. The finding that ICT demands was one of the factors with more robust associations to cognitive complaints, primarily cross-sectionally but also prospectively, is not surprising given that high ICT demands (by definition) affect the work situation in ways that may have direct implications for cognitive work performance. High ICT demands in terms of multiple and frequent phone calls and new e-mails that require handling (processing information and responding appropriately) are likely to interrupt other ongoing tasks. This means that the employees may experience cognitive overload (there is more information and a larger number of concomitant work tasks than can be kept in mind at the same time). They may also have to engage in frequent task switching that requires repeated refocusing while monitoring distracting information (i.e. previous tasks that linger in mind and the expectancy of new incoming requests that may also require prompt handling). These types of tasks tap cognitive processes (referred to as executive cognitive functions) that are known to be cognitively costly and resource limited. This means that they take time and effort and are fatigable. There are for example cognitive experimental studies on fatigue of executive cognitive processes and costs of shifting attention [75], [76]. That is, these cognitive processes are prone to fatigue which involves performance decline that the individual may notice in terms of cognitive failures (i.e. mistakes). ICT demands may also involve a lower predictability of work and work load from day to day, or moment to moment, and reduced perceived possibilities to utilize own planning and control over the execution of work tasks. Overall predictability of the work situation has previously been found to be related to cognitive complaints among employees [46]. The boundary-less nature of work included in the measure of ICT demands may also involve a work situation that accommodates extended hours of uncompensated work when performance demands are high, thus potentially reducing time for private life and recuperation [77].

As high ICT demands likely involve the components described above, this may also entail frequent and prolonged heightening of arousal in order to cope with the cognitive demands (e.g. [78]). For some individuals this could result in transient negative effects on cognitive functioning (executive cognitive functioning in particular) from supra-optimal sympathetic arousal in the short term (e.g. [79]), as discussed above.

The hypothesized reduction in likelihood of cognitive complaints from having good material resources and being qualified for the job was confirmed and supports the importance of having sufficient resources for the job posited in the job demands-resources model, also with regard to cognitive complaints.This may be because employees with more material resources and qualifications for the job have to engage in fewer compensatory activities and concerns that would demand extra cognitive effort and thus occupy cognitive resources that are limited.

Emotional demands unexpectedly reduced the likelihood of cognitive complaints. The extent to which there are job resources matching the emotional demands should be important in determining if emotional demands [80] have a positive effect on cognitive complaints. However, crude correlations between emotional demands and social support, as well as with resources at work are in fact negative. This, together with the fact that the associations are very small, caution us from making any further interpretations of this possible chance finding.

High decision authority was only weakly protective against cognitive complaints cross-sectionally in this study. This is similar to Danish population studies, finding that low decision authority is related to cognitive stress symptoms (complaints) in men but not in women cross-sectionally [44]. This gender difference was not seen in our results, but similar results have been found in SLOSH in relation to depressive symptoms [68]. In most population studies men have also reported a higher level of decision authority at work than women [37]. The health benefits of high decision authority may also depend on other factors. For example, a very high workload [81] as well as unclear goals and work roles can decrease the possibility to utilize structures for exerting control.

In our study, past and present conflicts were more strongly associated with cognitive complaints cross-sectionally in women than in men. However, when adjusting for depression, the higher association in women decreased, suggesting that depressive symptoms are more comorbid with- and possibly a mediator of cognitive complaints in women when there are conflicts at work. This may partly be explained by the generally higher prevalence of depressive symptoms among women [58], [82].

A clear gender interaction was also observed in the prospective results wherein quantitative demands were consistently more strongly associated with future cognitive complaints in women than in men. Only in women were quantitative demands a clear predictor of future cognitive complaints in all models, including after adjustment for baseline cognitive complaints.

There are multiple possible reasons for this gender difference. The fact that women have a higher work load in private life [83] can play an important role in magnifying the risk for aversive effects from high quantitative job demands [84], [85], as well as possibly limiting the benefits of other potentially protecting factors like job decision authority. The stronger relation between high demands and cognitive complaints among women could be due both to a higher total work load of paid and unpaid work, as well as role-conflicts concerning work versus private life priorities in day-to-day life [86], which should also be considered in future work.

The characteristics of a work situation associated with high job demands could also differ between genders in ways that are important for the actual work load.

For example, qualitative studies on women with high job demands and job-stress related exhaustion have shown a pattern that differentiates the situation of these white-collar women in relatively high positions from their male colleagues [87]. In addition to a higher work-load in private life, these women also tend to get a higher load at work from getting and taking on extra (-role) tasks, as well as even performing others' tasks, as there is a perception that this is expected and required of them to keep the job [87].

Furthermore, women have repeatedly been found to be rated as less qualified than male counterparts with exactly the same qualifications (e.g. [88]), suggesting that there is a basis for different levels of achievement pressure between men and women.

Thus, there may be other differences in the psychosocial work environment of men and women that contribute to differential perceptions- and types of work load with differential consequences in terms of symptom levels (such as cognitive complaints).

It is worth noting that almost twice as many women as men in the work force report high levels of cognitive complaints, as well as depressive symptoms. Differential working conditions and interpretation frames of performance expectations which could increase the perceived workload, as well as a combination of perceived high job demands and a higher work load in private life may contribute to this pattern in women. Further studying the distribution and effects of more qualitatively and quantitatively specific types of gendered job support, demands and expectations from superiors and colleagues, would be useful to further map the psychosocial pathways leading to health problems like cognitive complaints in men and women, respectively [80], [83], [86].

Several factors were only- or were more strongly- associated to cognitive complaints cross-sectionally. This suggests that work factors are more likely to exert their effects on cognitive complaints acutely, rather than cause chronic effects on subjective cognitive functioning per se that persist over longer time. Thus, cognitive complaints may in the majority of cases indicate current unfavorable working conditions.

As mentioned in the introduction, the present study only permits inferences to be made regarding subjective cognitive complaints, as no measures of objective cognitive performance were used and associations between subjective cognitive complaints and cognitive performance measures are generally weak at a group level.

Thus, the subjective assessment of cognitive complaints does not reveal what cognitive complaints represent in this sample. Future work should investigate the significance of subjective cognitive complaints in terms of actual cognitive functioning in the general working population as such studies are still limited. Cognitive complaints in the present study may represent accurate observations of cognitive failures and a discrepancy between available cognitive resources and current cognitive demands, but the reasons for the cognitive failures may be cognitive overload and temporary depletion of cognitive resources, or fatigue, rather than more chronically impaired cognitive functioning per se. Cognitive complaints may also represent accurate observations of acute neuropsychological stress effects (via various mechanisms [79]) on cognitive function, impairing especially prefrontal cortical dependent executive functions important for cognitive activities such as memory retrieval, decision making, concentration and clear thinking. Indeed, the occurrences of momentary stressful events have been associated to corresponding momentary increases in secretion of the stress hormone cortisol [89]. The aforementioned possible reasons for the experience of cognitive complaints all represent effects that could plausibly be elicited by certain work factors. Conscious or subconscious cognitive appraisal [90] of situations at work is likely to heavily influence whether certain factors elicit stress responses that in turn can have aversive effects on certain cognitive functions [79]. However, frequent and long lasting experience of work stressors related to cognitive complaints could potentially constitute a risk for more chronic and prolonged effects on cognitive functioning due to chronic elevation of physiological stress levels, which may cause impaired neurogenesis and structural brain damage such as dendritic retraction (animal studies- see e.g. [91]), as well as lead to HPA dysregulation with dampened cortisol levels over time [11], [13]. Dampened cortisol, the experience of cognitive complaints, as well as impairments in cognitive function are all implicated in chronic stress syndromes. While elevated stress levels can cause acute aversive effects on certain cognitive functions, negative effects on cognitive functioning could potentially generate more stress via appraisal processes and decreased coping ability. This way, a vicious circle of cognitive symptoms and more stress could arise that over time could potentially lead to more chronic effects on subjective as well as objective cognitive function in more extreme cases.

Now, this study provides the most support for quantitative demands, ICT demands and social support as predictors of cognitive complaints over a two year period. “Quantitative job demands” corresponds to the general workload and thus it is plausible that this may have more long lasting effects on cognitive health. This may be due to that this factor possibly captures more chronic workload exposure, as seen in long term effects on physiological [92], [93] and psychological [38], [55] health measures, as well as the cognitive symptoms and functional impairments seen in people with chronic job-/stress related exhaustion or burnout [7], [8], [9], [10], [11], [12], [13]. However, properly studying the causality in relations between work factors and cognitive complaints would require more than two measurement points, preferably closer in time, to assess which type of work factors and exposure durations that affect cognitive complaints in a more transitory versus a more permanent way. For example, acute psychosocial stressors have been shown to impact executive cognitive functioning and functional brain activation [17], [18], [19]. Furthermore, four weeks of heightened psychosocial stress has been shown to negatively impact on prefrontal cortex function and prefrontal-parietal functional connectivity during an executive cognitive performance task (fMRI during a set shifting task), but these effects were reversible after four weeks of low stress in the same subjects [16]. This shows the possibly transient nature of effects on cognitive functioning, going from a period of high strain with associated reduced executive function to a period of reduced strain and subsequent reversal of the negative effects on cognitive function. Similarly, follow-up studies of chronic stress patients with cognitive complaints have shown improvements in cognitive functioning after a period of rehabilitation [94]. It is also possible that there are bi-directional relationships or reverse causation in some associations between work factors and cognitive complaints. This means that some work features may be experienced as more demanding if a person has for example concentration problems. Similar suggestions have been made elsewhere [10], [95], [96].

These different possibilities should be investigated further in future studies with more measurement points and analyses of both causal directions.

The inclusion of depressive symptoms and sleeping problems in models of the relationships between work factors and cognitive complaints adds to the understanding of this field as the few existing epidemiological studies of work factors and cognitive complaints have not included the role of these factors.

The current results showed that depressive symptoms was a strong predictor of cognitive complaints both cross-sectionally and prospectively, and played a role in all of the significant relationships between the respective work factors and cognitive complaints, although baseline cognitive complaints explained most of the variance in cognitive complaints at follow-up. Prior working conditions only explained a very small portion of future cognitive complaints levels among employees, after taking prior depressive symptoms or prior cognitive complaints into account.

This means that subjects with a high level of depressive symptoms also have a higher risk of experiencing cognitive complaints and the working conditions associated with cognitive complaints, especially poor social support. Sleeping problems also played a role in the relationships between work factors and cognitive complaints but not to the same extent as depressive symptoms.

### Strengths and limitations of the study

The current study samples from SLOSH were large in size and nationally representative of the Swedish working population which strengthens the generalizability of the results. The current study allowed for analyses of the relationships between psychosocial work factors and cognitive functioning while also adjusting for a wide range of demographic, health and lifestyle factors in order to avoid ‘spurious’ relationships and investigate the role of potentially mediating factors in the observed associations.

As the current results are based on cross-sectional and prospective data, they provide an indication that certain factors at work may play an important role for the level of cognitive complaints in employees and that most of these work factors may play a role primarily for concurrent cognitive complaints while few work factors also play a role for future cognitive complaints. Such differential relationships that depend on time interval may be due both to the nature of the effect that a specific factor has on perceived cognitive functioning as well as how stable the specific work factor is in terms of exposure intensity and duration over longer time periods.

The cross-sectional study design does not allow for causal inferences or investigation of the potential mediation of sleep and/or depression since this would require a longitudinal design with more measurement points. Thus, the observed relationships could represent different causal paths, e.g.: 1) psychosocial factors causing cognitive complaints, 2) cognitive complaints causing the experience of certain psychosocial work factors as greater or lesser in magnitude, or 3) the relationship is explained by other underlying factors that are affecting ratings of both work factors and cognitive complaints.

However, the prospective analyses indicate that certain work factors such as quantitative job demands may increase levels of cognitive complaints, especially since this factor was associated with future cognitive complaints both without and with adjustment for baseline cognitive complaints.

There is also a possibility that common method bias influence the results since all measures are self-reported. For example, state or trait mood or affectivity effects could inflate relationships between the self-report measures being studied.

In addition, as stated earlier, some studies have found that cognitive complaints are more related to negative affect, than to actual deficits in cognitive function. However, the inclusion of depressive symptoms in one step of the sequential regression analysis should also provide adjustment for effects that are attributable to negative affectivity, especially considering that the main item (with the highest factor loading) in the adopted depression scale is low mood. Depression has been found to be particularly and highly correlated with negative affect elsewhere (e.g. [97]).

Thus, it seems reasonable that the inclusion of the current depression scale largely will have adjusted for the common variance in work factors and cognitive complaints that is attributable to negative affect.

Furthermore, the use of broad measures of some aspects of the psychosocial work environment makes it possible that the intended dimension is not well captured. For example the self-rated qualification level for the current job may capture other psychological dimensions such as job self-efficacy rather than the objective qualification level in relation to the job. That is, it may indicate a person's perceptions of own abilities to perform the job, rather than actual abilities. Job self-efficacy in turn may be related to other factors than actual abilities to perform the job and has for example been related to the leadership style and support of superiors [98], [99], [100].

Although there are many questions that remain to be answered, the current study implicates some work environment factors as playing an important role in levels of cognitive complaints, which could guide further studies and systematic work environment design to optimize individuals' psychosocial occupational environment with regard to subjective cognitive functioning.

Further longitudinal studies with more measurement points and statistical methods where changes in the predictors and outcome over time, as well as measurement errors, can be taken into account in a more precise way will provide a better basis from which causal inferences can be made.

## Conclusions

Overall, the study indicated that the psychosocial work environment is associated with cognitive complaints in the Swedish working population, most strongly so cross-sectionally.

The role of psychosocial work factors in cognitive complaints should thus be taken into account in systematic work environment development and management to ensure optimal working conditions for all employees with regard to subjective cognitive functioning.

The findings suggest that employees' general work load, as well as routines and expectations regarding the handling of ICT demands are factors to consider in efforts to prevent or reduce cognitive complaints among employees.

The findings could guide health care workers, managers as well as individuals with cognitive complaints to better understand the possible causes of- and remedies for the experience of cognitive problems.

## Supporting Information

Table S1 **Cross sectional study sample (2008) characteristics.** Demographic and potential confounders.
(DOC)

Table S2 **Cognitive complaints and work characteristics for the cross sectional study sample.** N = 9756.
(DOC)

Table S3 **Correlation coefficients between measures 1–15.** Cross sectional study sample (2008/T2).
(DOC)

Table S4 **Cross sectional study results (2008).** n = 8362. Standardized β coefficients and adjusted R2 for multiple regression models 1–4, with cognitive complaints as the outcome.
(DOC)

Table S5 **Prospective study results (T1–T2/2006–2008).** n = 3264. Standardized β coefficients and adjusted R2 for multiple regression models 1–4 with predictors at T1 (2006) and cognitive complaints score (1–5) at T2 (2008) as the outcome.
(DOC)
