# Supplementary material for: Psychosocial Working Conditions and Cognitive Complaints among Swedish Employees
Source: PLoS One. 2013 Apr 1;8(4):e60637. doi: 10.1371/journal.pone.0060637 (PMC3613346; doi:10.1371/journal.pone.0060637)
Supplement: Table S1 — Cross sectional study sample (2008) characteristics. Demographic and potential confounders. (DOC) [file pone.0060637.s001.doc]

| Table S1. Cross sectional study sample (2008) characteristics. Demographic and potential confounders. | | | | | |
| --- | --- | --- | --- | --- | --- |
| Measure | % of sample | % Missing | Mean | Std. Dev. | Min-Max |
| Sex: | ∙ | .0 | ∙ | ∙ | ∙ |
| *Male* | 45.7 | ∙ | ∙ | ∙ | ∙ |
| *Female* | 54.3 | ∙ | ∙ | ∙ | ∙ |
| Age | ∙ | .0 | 48.4 | 10.63 | 20-70 |
| Education: | ∙ | .1 | ∙ | ∙ | ∙ |
| *No upper secondary* | 10,3 | ∙ | ∙ | ∙ | ∙ |
| *Upper secondary* | 46.3 | ∙ | ∙ | ∙ | ∙ |
| *Univ.studies < 2 years* | 6.4 | ∙ | ∙ | ∙ | ∙ |
| *Univ. studies ≥ 2 years* | 37 | ∙ | ∙ | ∙ | ∙ |
| Yearly income (1000’s SKR) | 305.92 | .0 | 305.65 | 154.99 | 0-4128 |
| CVD, (incidence) | 2.7 | 4.0 | ∙ | ∙ | ∙ |
| Non-specific psych. illness (incidence) | 3.2 | 3.3 | ∙ | ∙ | ∙ |
