# Supplementary material for: Psychosocial Working Conditions and Cognitive Complaints among Swedish Employees
Source: PLoS One. 2013 Apr 1;8(4):e60637. doi: 10.1371/journal.pone.0060637 (PMC3613346; doi:10.1371/journal.pone.0060637)
Supplement: Table S2 — Cognitive complaints and work characteristics for the cross sectional study sample. N = 9756. (DOC) [file pone.0060637.s002.doc]

| Table S2. Cognitive complaints and work characteristics for the cross sectional study sample. N=9756 | | | | | | | |
| --- | --- | --- | --- | --- | --- | --- | --- |
| Measure | % | % Missing | Mean | Std. Dev. | Min-Max | Cronbach’s alpha | Auto-correlation T1-T2*** |
| Quantitative demands |  | .5 | 2.68 | .52 | 1-4 | .72 | .60 |
| Skill discretion | ∙ | .5 | 3.09 | .46 | 1-4 | .59 | .67 |
| Decision authority | ∙ | 1.1 | 3.19 | .70 | 1-4 | .73 | .60 |
| ICT demands | ∙ | 1.4 | 2.99 | .80 | 1-5 | .87 | .64 |
| Emotional demands | ∙ | 1 | 2.74 | .87 | 1-4 | .78 | .76 |
| Social support | ∙ | 2.1 | 3.11 | .52 | 1-4 | .86 | .54 |
| Resources | ∙ | .7 | 3.51 | .53 | 1-4 | .71 | .29 |
| Perceived qualification | ∙ | .8 | ∙ | ∙ | ∙ | ∙ | ∙ |
| *Qualified* | 56.4 | ∙ | ∙ | ∙ | ∙ | ∙ | ∙ |
| *Underqualified* | 18.2 | ∙ | ∙ | ∙ | ∙ | ∙ | .35 |
| *Overqualified* | 25.4 | ∙ | ∙ | ∙ | ∙ | ∙ | .46 |
| Conflicts at work, during past 2 years | ∙ | 2.7 | ∙ | ∙ | ∙ | ∙ | ∙ |
| *No conflicts* | 49.1 | ∙ | ∙ | ∙ | ∙ | ∙ | ∙ |
| *Conflicts, finished* | 38.2 | ∙ | ∙ | ∙ | ∙ | ∙ | ∙ |
| *Conflicts, ongoing* | 12.7 | ∙ | ∙ | ∙ | ∙ | ∙ | ∙ |
| Depressive symptoms | ∙ | 1.3 | 1.94 | .88 | 1-5.4 | .91 | .53 |
| Disturbed sleep (high degree) | 16.8 | 2.3 | ∙ | ∙ | 0-1 | ∙ | .44 |
| Awakening problems (high degree) | 16.6 | 2.1 | ∙ | ∙ | 0-1 | ∙ | .46 |
| Cognitive complaints index: | ∙ | .8 | 2.16 | .82 | 1-5 | .91 | .66 |
| *Never-Seldom (1-2)* | 51.6 | ∙ | ∙ | ∙ | ∙ | ∙ | ∙ |
| *Seldom-Sometimes (2.25-3)* | 37.3 | ∙ | ∙ | ∙ | ∙ | ∙ | ∙ |
| *Sometimes-Often (3.25-4)* | 10.3 | ∙ | ∙ | ∙ | ∙ | ∙ | ∙ |
| *Often-Always (4.25-5)* | .8 | ∙ | ∙ | ∙ | ∙ | ∙ | ∙ |
| Concentration: | ∙ | .8 | 2.32 | .94 | 1-5 | ∙ | .58 |
| *Never-Seldom (1-2)* | 57.3 | ∙ | ∙ | ∙ | ∙ | ∙ | ∙ |
| *Sometimes (3)* | 32.6 | ∙ | ∙ | ∙ | ∙ | ∙ | ∙ |
| *Often-Always (4-5)* | 10.1 | ∙ | ∙ | ∙ | ∙ | ∙ | ∙ |
| Making decisions: | ∙ | .9 | 2.14 | .87 | 1-5 | ∙ | .54 |
| *Never-Seldom (1-2)* | 67.5 | ∙ | ∙ | ∙ | ∙ | ∙ | ∙ |
| *Sometimes (3)* | 26.9 | ∙ | ∙ | ∙ | ∙ | ∙ | ∙ |
| *Often-Always (4-5)* | 5.5 | ∙ | ∙ | ∙ | ∙ | ∙ | ∙ |
| Memory: | ∙ | .9 | 2.11 | .97 | 1-5 | ∙ | .58 |
| *Never-Seldom (1-2)* | 66.3 | ∙ | ∙ | ∙ | ∙ | ∙ | ∙ |
| *Sometimes (3)* | 25.2 | ∙ | ∙ | ∙ | ∙ | ∙ | ∙ |
| *Often-Always (4-5)* | 8.5 | ∙ | ∙ | ∙ | ∙ | ∙ | ∙ |
| Thinking clearly: | ∙ | .8 | 2.07 | .91 | 1-5 | ∙ | .57 |
| *Never-Seldom (1-2)* | 68.7 | ∙ | ∙ | ∙ | ∙ | ∙ | ∙ |
| *Sometimes (3)* | 25.2 | ∙ | ∙ | ∙ | ∙ | ∙ | ∙ |
| *Often-Always (4-5)* | 6.1 | ∙ | ∙ | ∙ | ∙ | ∙ | ∙ |
| *** For all correlations p<0.001. Correlations are for the prospective study sample. | | | | | | | |
