# Supplementary material for: Psychosocial Working Conditions and Cognitive Complaints among Swedish Employees
Source: PLoS One. 2013 Apr 1;8(4):e60637. doi: 10.1371/journal.pone.0060637 (PMC3613346; doi:10.1371/journal.pone.0060637)
Supplement: Table S3 — Correlation coefficients between measures 1–15. Cross sectional study sample (2008/T2). (DOC) [file pone.0060637.s003.doc]

| Table S3. Correlation coefficients between measures 1-15. Cross sectional study sample (2008/T2). | | | | | | | | | | | | | | | |
| --- | --- | --- | --- | --- | --- | --- | --- | --- | --- | --- | --- | --- | --- | --- | --- |
|  | 1. | 2. | 3. | 4. | 5. | 6. | 7. | 8.^†^ | 9.^†^ | 10.^†^ | 11.^†^ | 12. | 13.^†^ | 14.^†^ | 15. |
| 1. | 1 | ∙ | ∙ | ∙ | ∙ | ∙ | ∙ | ∙ | ∙ | ∙ | ∙ | ∙ | ∙ | ∙ | ∙ |
| 2. | .190** | 1 | ∙ | ∙ | ∙ | ∙ | ∙ | ∙ | ∙ | ∙ | ∙ | ∙ | ∙ | ∙ | ∙ |
| 3. | -.092** | .289** | 1 | ∙ | ∙ | ∙ | ∙ | ∙ | ∙ | ∙ | ∙ | ∙ | ∙ | ∙ | ∙ |
| 4. | .382** | .257** | .070** | 1 | ∙ | ∙ | ∙ | ∙ | ∙ | ∙ | ∙ | ∙ | ∙ | ∙ | ∙ |
| 5 | .304** | .288** | .104** | .222** | 1 | ∙ | ∙ | ∙ | ∙ | ∙ | ∙ | ∙ | ∙ | ∙ | ∙ |
| 6. | -.283** | .137** | .245** | -.091** | -.028** | 1 | ∙ | ∙ | ∙ | ∙ | ∙ | ∙ | ∙ | ∙ | ∙ |
| 7. | -.434** | -.047** | .118** | -.199** | -.209** | .310** | 1 | ∙ | ∙ | ∙ | ∙ | ∙ | ∙ | ∙ | ∙ |
| 8.^†^ | .029** | .050** | -.042** | .046** | .042** | -.004 | -.050** | 1 | ∙ | ∙ | ∙ | ∙ | ∙ | ∙ | ∙ |
| 9.^†^ | .080** | -.029** | -.046** | .025* | .001 | -.118** | -.089** | -.276** | 1 | ∙ | ∙ | ∙ | ∙ | ∙ | ∙ |
| 10.^†^ | .124** | .073** | .012 | .112** | .165** | -.048** | -.087** | .032** | .048** | 1 | ∙ | ∙ | ∙ | ∙ | ∙ |
| 11.^†^ | .163** | .015 | -.079** | .063** | .125** | -.261** | -.175** | .029** | .080** | -.300** | 1 | ∙ | ∙ | ∙ | ∙ |
| 12. | .299** | -.043** | -.140** | .193** | .119** | -.325** | -.276** | .089** | .054** | .077** | .185** | 1 | ∙ | ∙ | ∙ |
| 13.^†^ | .133** | -.002 | -.047** | .096** | .066** | -.146** | -.113** | .042** | .044** | .027* | .093** | .322** | 1 | ∙ | ∙ |
| 14.^†^ | .151** | .003 | -.073** | .098** | .021* | -.162** | -.134** | .042** | .056** | .022* | .112** | .354** | .523** | 1 | ∙ |
| 15. | .325** | .024* | -.101** | .269** | .150** | -.280** | -.276** | .125** | .002 | .106** | .106** | .600** | .294** | .315** | 1 |
| 1. Quantitative demands, 2. Skill discretion, 3. Decision authority, 4. ICT demands, 5. Emotional demands, 6. Social support, 7. Resources, 8. Underqualified, 9. Overqualified, 10. Conflicts, finished, 11. Conflicts, ongoing, 12. Depression, 13. Disturbed sleep, 14. Awakening problems, 15. Cognitive complaints.  * p<.05. ** p<.01. *** p<.001.  †Spearman correlation coefficient. All else are Pearson correlation coefficients. | | | | | | | | | | | | | | | |
