# Supplementary material for: Psychosocial Working Conditions and Cognitive Complaints among Swedish Employees
Source: PLoS One. 2013 Apr 1;8(4):e60637. doi: 10.1371/journal.pone.0060637 (PMC3613346; doi:10.1371/journal.pone.0060637)
Supplement: Table S4 — Cross sectional study results (2008). n = 8362. Standardized β coefficients and adjusted R2 for multiple regression models 1–4, with cognitive complaints as the outcome. (DOC) [file pone.0060637.s004.doc]

| Table S4. Cross sectional study results (2008). n=8362.  Standardized β coefficients and adjusted R^2^ for multiple regression models 1-4, with cognitive complaints as the outcome. | | | | |
| --- | --- | --- | --- | --- |
| Measure | *1* | *2* | 3 | *4* |
| Quantitative demands | .14*** | .15*** | .08*** | .08*** |
| Skill discretion | -.04*** | -.04** | .00 | -.01 |
| Decision authority | -.03** | -.01 | .00 | .00 |
| IT demands | .17*** | .17*** | .12*** | .12*** |
| Emotional demands | .02* | -.01 | -.02* | -.01 |
| Social support | -.15*** | -.16*** | -.06*** | -.05*** |
| Resources | -.11*** | -.10*** | -.05*** | -.05*** |
| Qualified (reference) | ∙ | ∙ | ∙ | ∙ |
| Underqualified | .09*** | .08*** | .05*** | .05*** |
| Overqualified | -.03** | -.03*** | -.04*** | -.04*** |
| No conflicts (reference) | ∙ | ∙ | ∙ | ∙ |
| Conflicts. finished | .07*** | .06*** | .05*** | .05*** |
| Conflicts. ongoing | .09*** | .07*** | .04*** | .03*** |
| Depression | ∙ | ∙ | .48*** | .44*** |
| Disturbed sleep | ∙ | ∙ | ∙ | .06*** |
| Awakening problems | ∙ | ∙ | ∙ | .09*** |
| Accumulated adjusted R^2^ | .199 | .238 | .420 | .433 |
| 1. Psychosocial work factors, unadjusted model.  2. Adjusted for Age, Sex, Educational level, Income, Alcohol consumption, Cardiovascular disease, Psychiatric illness.  3. Adjusted for Depressive symptoms, in addition to model 2 covariates.  4. Adjusted for Disturbed sleep and Awakening problems, in addition to model 3 covariates.  * p<.05. ** p<.01. *** p<.001. | | | | |
