# Supplementary material for: Psychosocial Working Conditions and Cognitive Complaints among Swedish Employees
Source: PLoS One. 2013 Apr 1;8(4):e60637. doi: 10.1371/journal.pone.0060637 (PMC3613346; doi:10.1371/journal.pone.0060637)
Supplement: Table S5 — Prospective study results (T1–T2/2006–2008). n = 3264. Standardized β coefficients and adjusted R2 for multiple regression models 1–4 with predictors at T1 (2006) and cognitive complaints score (1–5) at T2 (2008) as the outcome. (DOC) [file pone.0060637.s005.doc]

| Table S5. Prospective study results (T1-T2/2006-2008). n=3264. Standardized β coefficients and adjusted R^2^ for multiple regression models 1-4 with predictors at T1 (2006) and cognitive complaints score (1-5) at T2 (2008) as the outcome. | | | | | | | | |
| --- | --- | --- | --- | --- | --- | --- | --- | --- |
| Measure | *1* | *2* | *3* | *4* | *1^a^* | *2^a^* | *3^a^* | *4^a^* |
| Quantitative demands | .14*** | .15*** | .09*** | .08*** | .05** | .05** | .04** | .04** |
| Skill discretion | -.06** | -.04* | -.02 | -.02 | -.02 | -.02 | -.02 | -.02 |
| Decision authority | .00 | .02 | .04* | .04* | .01 | .02 | .02 | .02 |
| ICT demands | .13*** | .12*** | .09*** | .08*** | .03* | .03* | .03* | .03* |
| Emotional demands | .06*** | .02 | .02 | .02 | .03 | .01 | .01 | .01 |
| Social support | -.15*** | -.16*** | -.05** | -.05** | -.03* | -.03* | -.01 | -.01 |
| Resources | -.06*** | -.06*** | -.01 | -.01 | -.02 | -.02 | -.01 | -.01 |
| Underqualified^‡^ | .08*** | .08*** | .05** | .05** | .04** | .04** | .03* | .03* |
| Overqualified^‡^ | .00 | .00 | -.02 | -.02 | .00 | .00 | -.01 | -.01 |
| Depression | ∙ | ∙ | .45*** | .43*** | ∙ | ∙ | .15*** | .14*** |
| Disturbed sleep | ∙ | ∙ | ∙ | .04* | ∙ | ∙ | ∙ | .02 |
| Awakening problems | ∙ | ∙ | ∙ | .04* | ∙ | ∙ | ∙ | .00 |
| Cognitive complaints at T1 | ∙ | ∙ | ∙ | ∙ | .62*** | .61*** | .53*** | .53*** |
| Adjusted R^2^ | .121 | .144 | .306 | .310 | .442 | .448 | .459 | .459 |
| 1. Psychosocial work factors at T1, unadjusted model.  2. Adjusted for Age, Sex, Educational level, Income, Alcohol consumption, Cardiovascular disease and Psychiatric illness at T1.  3. Adjusted for Depressive symptoms at T1, in addition to model 2 covariates.  4. Adjusted for Disturbed sleep and Awakening problems at T1, in addition to model 3 covariates.  ^a^ Cognitive complaints at T1 has been adjusted for in addition to the other specified measures in the respective models.  ^‡^Reference: qualified.  * p<.05. ** p<.01. *** p<.001. | | | | | | | | |
